# *CYP2D6* Genotype-Based Dose Recommendations for Risperidone in Asian People

**DOI:** 10.3389/fphar.2020.00936

**Published:** 2020-08-04

**Authors:** Yuanxia Cui, Hao Yan, Yi Su, Lifang Wang, Tianlan Lu, Dai Zhang, Weihua Yue

**Affiliations:** ^1^Institute of Mental Health, Peking University Sixth Hospital, Beijing, China; ^2^National Clinical Research Center for Mental Disorders & Key Laboratory of Mental Health, Ministry of Health (Peking University) & Chinese Academy of Medical Sciences Research Unit (No.2018RU006), Beijing, China; ^3^PKU-IDG/McGovern Institute for Brain Research, Peking University, Beijing, China; ^4^School of Nursing, Peking University, Beijing, China

**Keywords:** cytochrome P-450, *CYP2D6*, polymorphism, pharmacogenetics, risperidone

## Abstract

The aim of this study was to provide dose recommendations for risperidone in Asian people based on cytochrome P450 enzyme *CYP2D6* genotype. First, we investigated the influence of *CYP2D6* polymorphism on the pharmacokinetics of risperidone in Chinese patients with schizophrenia. Then, we performed a search for studies covering the relationship between pharmacokinetic parameters of risperidone and *CYP2D6* genotype. Pooled pharmacokinetic parameters were meta-analyzed using a random-effects model. Lastly, we calculated the dose adjustment for risperidone based on *CYP2D6* genotype for white and Asian people. Significant differences between the extensive metabolizer and intermediate metabolizer groups were observed for dose-adjusted risperidone level, 9-hydroxyrisperidone level, and risperidone/9-hydroxyrisperidone ratio, but not for the total active moiety. Meta-analysis showed that significant differences were observed among the four phenotype groups, including steady state concentration, peak risperidone concentration, and the area under the curve, using the Kruskal-Wallis test. No differences were found in oral clearance. For risperidone, dose recommendations for poor and ultrarapid metabolizers of *CYP2D6* for Asians were different compared to that for white people for poor metabolizers (dose adjustment around 45% for white people, while for Asians the risperidone dose should be reduced by 26%). For ultrarapid metabolizers, risperidone dose should be increased by about 33% for white people and 30% for Asians. This was a first attempt to apply pharmacogenetics to suggest dose-regimens for Asian people; further research to replicate and extend these findings is recommended.

## Introduction

Risperidone (RIS) is an atypical antipsychotic (AAP) drug that is prescribed for the treatment of autism, schizophrenia, and acute bipolar mania, which is metabolized by the CYP2D6 enzyme in the liver to its major active metabolite, 9-hydroxyrisperidone (9-OH-RIS, also known as paliperidone). Over 100 allelic variants of the *CYP2D6* gene have been reported (https://www.pharmvar.org/gene/CYP2D6). These give rise to four different metabolizer phenotypes (enzyme activity levels) based on the sum of an activity score that is attributed to each allele including: (1) poor metabolizers (PM) without enzyme activity (activity score 0); (2) intermediate metabolizers (IM) with reduced enzyme activity (activity score 0–1.25); (3) normal metabolizers (NM) with normal activity (activity score 1.25–2.25); and (4) ultrarapid metabolizers (UM) with increased enzyme activity (activity score >2.25) (Caudle et al., 2020). Although it was believed that the sum of the plasma risperidone and 9-hydroxyrisperidone concentration (known as the “active moiety”) is responsible for the therapeutic response to, and adverse effects of, risperidone, there are prior data that indicate switching from risperidone to another drug that is higher in UMs and PMs (Jukic et al., 2019). The plasma concentration of risperidone may range from subtherapeutic levels in the UM group to supratherapeutic and potentially toxic concentrations in the PM group, thereby (if RIS and 9-OH-RIS differ in adverse effect profile) potentially increasing the possibility of adverse effects.

In previous studies, the plasma concentration of risperidone and the risperidone/9-hydroxyrisperidone ratio in patients varied by *CYP2D6* predicted phenotype group, suggesting that the determination of an accurate *CYP2D6* genotype-predicted phenotype may be helpful for the individualization of drug therapy in a clinical setting (van der Weide and van der Weide, 2015; Vanwong et al., 2016).

Risperidone dose adjustments were recommended based on *CYP2D6* metabolic phenotype (Kirchheiner et al., 2001; Kirchheiner et al., 2004; Kirchheiner et al., 2005). As significant differences in *CYP2D6* allele frequencies have been observed between different populations (Gaedigk et al., 2017), dose recommendations presented in previous studies may not be applicable to East Asian populations, such as the Chinese. In a study by Stingl et al. (Stingl et al., 2013), an updated version of pharmacogenetic-based therapeutic recommendations for risperidone was presented, integrating previous reviews (Kirchheiner et al., 2001; Kirchheiner et al., 2004; Kirchheiner et al., 2005) of studies on the pharmacogenetics of psychotropics that were published before 2010. In their analysis, nine studies were included (eight Caucasian, and one Korean study). Pharmacokinetic studies often have small sample sizes with limited precision in parameter estimates; it is therefore necessary to perform systematic reviews and meta-analyses to provide more robust estimates. We aimed to contribute to the data available on Asian people (specifically, Chinese) and to review data to date in order to develop risperidone dose recommendations for Asians.

## Methods

### Influence of *CYP2D6* Variants on the Pharmacokinetics of Risperidone

#### Participants

The outpatients who were followed up at Peking University Sixth Hospital (Beijing, China) were recruited in 2010-2011. In addition, the present study was a part of the Chinese Antipsychotics Pharmacogenomics Consortium (CAPOC) project (Yu et al., 2018). Individuals included in our study were diagnosed with schizophrenia based on the Structured Clinical Interview for DSM-IV, were of North Han Chinese ancestry, scored more than 60 on the Positive and Negative Syndrome Scale (PANSS) (and scored more than four on at least three positive items), were physically healthy, with all laboratory parameters within normal limits, had a condition that could be treated with oral medication, and were able to provide informed consent. Both first-episode and relapsed patients with schizophrenia were enrolled from inpatient departments of psychiatric hospitals affiliated with CAPOC. Patients were excluded from the study if they were diagnosed with schizoaffective disorder, delusional disorder, brief psychotic disorder, schizophreniform disorder, psychosis associated with substance use or medical conditions, learning disability, pervasive developmental disorder, delirium, dementia, amnesia, or other cognitive disorders. In addition, patients were excluded if they had severe, unstable physical diseases (such as diabetes, thyroid diseases, hypertension, and cardiac diseases), malignant syndrome or acute dystonia, documented histories of epilepsy or febrile convulsions, a DSM-IV diagnosis of alcohol or drug dependence, a history of drug-induced neuroleptic malignant syndrome, required long-acting injectable medication to maintain treatment adherence, were regularly treated with clozapine for treatment resistance during the past month (patients who had taken clozapine for reasons other than treatment resistance were eligible), were treated with electroconvulsive therapy (ECT) during the previous month, had previously attempted suicide, or had experienced symptoms of severe excitement and agitation, had abnormal liver or renal function (i.e., aspartate aminotransferase ≥80 U/L, alanine aminotransferase ≥80 U/L, blood urea nitrogen ≥9.75 mmol/L, urine creatinine ≥21.6 mmol per day), did not have a legal guardian (there was a hospital stipulation that written informed consent was required from the patient’s legal guardian), had QTc prolongation, a history of congenital QTc prolongation, or recent (i.e., within the past six months) myocardial infarction, were pregnant or breastfeeding women, or had a contraindication to any of the drugs to which they could be assigned. Patients who were already taking antipsychotic medications were obliged to switch to risperidone within one week, and adjusted drug dosages based on treatment effectiveness within two weeks (risperidone from 2 mg to 6 mg per day). The dosage then remained unchanged throughout the study. Patients receiving a risperidone-based regimen for more than four weeks were enrolled. Patients were excluded if they were unable to take medicine regularly or if they needed to change the medication. No combination of any other antipsychotics, antidepressants, antianxiety medications, or mood stabilizers was allowed during the study period. ECT therapy was prohibited. Participants were advised not to take drugs known to induce liver enzymes (e.g., rifampicin, carbamazepine, phenobarbital, phenytoin, etc.) for two weeks prior to enrollment and throughout the study period. Participants had to minimize usage of aspirin and non-steroidal anti-inflammatory drugs throughout the study period. Systematic psychotherapy was not permitted for the duration of the study. The study was approved by the institutional ethics review boards of the Peking University Sixth Hospital (Beijing, China), and written informed consent was obtained.

#### Sample Preparation and *CYP2D6* Genotyping

Blood samples were collected from a total of 130 patients after 4-6 weeks of treatment with a stable dose of risperidone, at 8.00 a.m., before the antipsychotic morning dose, 12 hours after the bedtime dose. Blood was collected in EDTA tubes for genotypic analysis and measurement of steady-state plasma concentrations of risperidone and 9-hydroxyrisperidone.

DNA was extracted using the Wizard Genomic DNA purification Kit (Promega). Genotyping was performed using the iPLEX™ Assay with a Sequenom MassARRAY^®^-SNP platform (CapitalBio Technology Co., Ltd.). The polymorphisms detected were as follows: *1708delT, 886C>T, 1022C>T, 1847G>A, 2540delAACT, 100C>T, 1612T>A, 2989G>A, 77G>A, 3202C>T, 882G>C, 2951G>C, 1977_1978insG, 2550delAl, 1759G>A, 2616delAAG, 2936A>C*, representing the following alleles: normal or uncertain function *1, *2,*43; decrease of function *9,*10, *14,*17, *41, *49; loss of function *3, *4, *6, *7, *11, *19, *20, *44, *56. *CYP2D6* gene copy number variation was derived from Illumina Human OmniZhongHua BeadChips, which were designed for the Chinese population, using the cnvpartition-cnv-analysis-plugin-v3.2.0 for GenomeStudio 2.0.

The CYP allele designations refer to those defined by the Pharmacogene Variation Consortium (https://www.pharmvar.org/gene/CYP2D6) (Gaedigk et al., 2019). The activity score was computed and the phenotype was extrapolated based on the genotypic data (**Supplement 1**, **Tables S1-1**, and **S1-2**).

#### Assay of Plasma Risperidone and 9-Hydroxyrisperidone Levels

The elimination half-lives of risperidone and 9-hydroxyrisperidone have been reported to be 3–29 h and 20–29 h, respectively (Huang et al., 1993; Yasui-Furukori et al., 2003). Therefore, plasma concentrations of these compounds had already reached a steady state in all participants before the blood samples were taken.

Plasma concentrations of risperidone and 9-hydroxyrisperidone were measured by a high-performance liquid chromatography tandem mass spectrometry coupled with an online solid-phase extraction method that could be used to measure risperidone, paliperidone, and olanzapine using small (40uL) samples as described (Ruan et al., 2018). The intra- and inter-assay coefficients of variation (CV) were 2.2% and 4.8% respectively. We first extracted the analytes from plasma samples and then pre-concentrated and purified it by C_8_ (5 μm, 2.1 × 30 mm) solid-phase extraction cartridges. Then the analytes were chromatographed on an Xbidge™ C_18_ column (3.5 μm, 100 × 2.1 mm), and analyzed by tandem mass spectrometry.

### Meta-Analysis of Pharmacokinetics Parameters of Risperidone

#### Literature Search and Search Strategy

We searched the following databases: PubMed, Embase, the Cochrane Library, the China National Knowledge Infrastructure (CNKI), and the Wanfang database, including all articles published up to Oct 8, 2019. The search strategy was based on combinations of the keywords “*CYP2D6*” AND “polymorphism” AND “risperidone OR ris.” Reference lists of retrieved articles or previous meta-analyses were manually checked to identify any further eligible studies.

#### Study Selection and Data Extraction

Two independent investigators screened titles and abstracts for relevance to the topic independently. The remaining potential publications were evaluated by reading the full text. Retrieved studies were included if they met the following criteria: 1) observational or clinical trial design, excluding abstracts and conference literature; 2) conducted in individuals (healthy volunteers or patients); 3) included measurements of the pharmacokinetic parameters of risperidone, such as the area under the curve (AUC), total clearance(Cl), and steady-state concentration (Css); and 4) determined *CYP2D6* polymorphism. Review articles and studies involving nonhuman participants and *in vitro* experiments were excluded. When the results of a study were reported in further interim analyses, only the most recent, complete, and updated data were included in this meta-analysis.

For each article, the following information was extracted from the article using a systematic extraction form: the first author’s name, year of publication, country or geographical origin of investigation, study design, follow-up, treatment, comparison, number of patients, and the outcome measurements. Any disagreements were resolved by discussion with a third investigator. In trials in which data were presented as the median (IQR) or median (range), we contacted the authors to request the raw data. In cases where we did not receive a response, the mean (SD) was calculated according to the 68–95–99.7 rule (Pukelsheim, 1994), also known as the Empirical rule, which states that the so-called three-sigma rule of thumb expresses a conventional heuristic that nearly all values may be taken to lie within three standard deviations of the mean. Even for non-normally distributed variables, at least 88.8% of cases should fall within a properly calculated three-sigma interval. For unimodal distributions, the probability of being within the three-sigma interval is at least 95% (Vysochanskij, 1980). The data in our meta-analysis were pharmacokinetic parameters adjusted by dosage, such as steady state dose-normalized plasma risperidone concentration (Css/dose), peak dose-normalized plasma risperidone concentration (Cmax/dose), and dose-normalized area under the plasma concentration (AUC/dose). If the extracted data were not presented as dose-adjusted, data were divided by the dose presented in the paper. For some raw data, the units were adjusted to facilitate consistency in the analysis.

#### Classification of Metabolizer Groups

Phenotypes, or the metabolizer group classification of *CYP2D6*, were assigned to participants depending on the combination of the two identified alleles. The “allele and gene activity score” system is one of the most popular classification systems (Caudle et al., 2020). **Table 1** shows the details of our data using this system. Individuals were classified into four different metabolic phenotypes according to predictive enzymatic activity, as described above. Based on the algorithm presented by Gaedigk et al. (2008, 2017) and Borges et al. (2006), participants were defined as normal metabolizers (NM) if two fully functional alleles were present or if they carried one reduced-function allele in combination with a fully functioning allele (AS:1.25-2.25), or intermediate metabolizers (IM) if they carried two reduced-function alleles or one fully functional and one nonfunctional allele, or one reduced and one nonfunctional allele (AS:0-1.25). In addition, poor metabolizers (PM) carried two nonfunctional alleles (AS=0); ultrarapid metabolizers (UM) were defined as having at least one extra copy of a functional allele (AS>2.25) (Caudle et al., 2020). No UMs or other structural variants were detected.

**Table 1:** The “allele and gene activity score” system.

*AS*	*Enzyme activity*	*ALLELE*
>1	increased	*1XN,*2XN
1	normal	*1,*2,*43
0.5/0.25^a^	reduced	*9,*10,*14,*17,*41,*44,*49
0	nonfunctional	*3,*4,*4XN,*5,*6,*7,*11, *19, *114,*20, *56

AS, activity score. ^a^an activity score of 0.25 is for *10.

### Statistical Analysis

#### The Influence of *CYP2D6* Polymorphism on the Steady-State Concentration of Risperidone

Data are presented as the median (IQR). Descriptive statistics were used to describe the clinical characteristics of all participants. After testing for normality, a Mann-Whitney U test was employed for between group comparisons. P values < 0.05 were considered nominally statistically significant (with no adjustment for multiple testing). Statistical analyses were performed using SPSS version 21.0 (SPSS Inc., Chicago, IL, USA).

#### Data Calculation for Dose Adjustment

Dose adjustments were computed according to a method described previously (Kirchheiner et al., 2004; Stingl et al., 2013). In brief, dose adjustments were based on differences in pharmacokinetic parameters, including oral clearance, area under the concentration time curve, and concentration at steady state, observed between phenotype groups.

For *CYP2D6*, about 0.5% of the Chinese population are poor metabolizers, 32.5% are intermediate, and 66.5% are normal metabolizers (Gaedigk et al., 2017). Thus, in general, the average dose (D_av_) that is recommended in Asian populations is as follows:

$${\text{D}}_{\text{av}}=0.005\;{\text{D}}_{\text{PM}}+0.33\;{\text{D}}_{\text{IM}}+0.665\;{\text{D}}_{\text{NM}}$$

where D_PM_, D_IM,_ and D_NM_ represent the optimal dose for the groups of poor metabolizers, intermediate metabolizers, and normal metabolizers, respectively.

In white people, about 7% are poor metabolizers of *CYP2D6*, 9% are intermediate, 82% normal metabolizers, and 2% ultrarapid metabolizers (Del Tredici et al., 2018). Thus, the average dose (D_av_) that is recommended in white people may be calculated as follows:

$${\text{D}}_{\text{av}}=0.07\;{\text{D}}_{\text{PM}}+0.09\;{\text{D}}_{\text{IM}}+0.82\;{\text{D}}_{\text{NM}}+0.02\;{\text{D}}_{\text{UM}}$$

It is hypothesized that the relationship between relative dose adjustments and relative changes of pharmacokinetic parameters observed in the phenotype groups is linear; therefore, dose adjustments for the intermediate and the poor metabolizer groups D_IM_ and D_PM_ were calculated according to equations 2 and 3 of Kirchheiner et al. (2004):

$$\begin{array}{l} {\text{D}}_{\text{IM}}={\text{D}}_{\text{NM}}\;\text{x}\;{\text{Cl}}_{\text{IM}}/{\text{Cl}}_{\text{NM}}\\ {\text{D}}_{\text{PM}}={\text{D}}_{\text{NM}}\;\text{x}\;{\text{Cl}}_{\text{PM}}/{\text{Cl}}_{\text{NM}} \end{array}$$

Assuming the average dose (Dav) is 100%, then the adjusted dose for the NM group, D_NM,_ for *CYP2D6* in Asian people is as follows:

$${\text{D}}_{\text{NM}}=100/(0.005{\text{Cl}}_{\text{PM}}/{\text{Cl}}_{\text{NM}}+0.330{\text{Cl}}_{\text{IM}}/{\text{Cl}}_{\text{NM}}+0.665),$$

where Cl_PM_, Cl_IM_, and Cl_NM_ are the empirically determined clearances for the respective phenotype groups.

For the computation of the dose adjustments in ultrarapid metabolizers (D_UM_) - if PK data were available - the percent dose was calculated as follows:

$${\text{D}}_{\text{UM}}{\text{=D}}_{\text{NM}}\;\text{x}\;{\text{Cl}}_{\text{UM}}/{\text{Cl}}_{\text{NM.}}$$

If no data on the UM group were available, the UM dose was computed based on the following linear relationship assumption:

$${\text{D}}_{\text{UM}}={\text{D}}_{\text{NM}}+({\text{D}}_{\text{NM}}-{\text{D}}_{\text{IM}})=2{\text{xD}}_{\text{NM}}-{\text{D}}_{\text{IM.}}$$

For AUC or concentration of steady state, the calculation of dose recommendations for each phenotype group was similar to the above.

#### Meta-Analysis

Statistical analysis was performed using STATA version 12.0 (StataCorp LLC, College Station, TX, USA). Due to substantial heterogeneity between studies, pooled pharmacokinetic parameters were calculated using a random-effects model. The inverse variance method was used for weighting studies. The heterogeneity between studies was formally assessed by the I^2^ statistic. Pre-planned subgroup analyses were conducted to examine whether effects of *CYP2D6* phenotype differed comparing Asian to white people. Comparison of the means of pooled pharmacokinetic parameters between metabolizer phenotype groups was performed by a Kruskal-Wallis test, as the data were not normally distributed, using GraphPad Prism 7.00 (GraphPad, La Jolla, CA, USA) software. A P-value of <0.05 was considered nominally significant.

## Results

### *CYP2D6* Genotype, Predicted Phenotype, and Its Effect on Risperidone Pharmacokinetics

The total number of north Han Chinese patients included in this study was 130, including 78 males and 52 females with schizophrenia aged 30.6 (18-46) years old, not known to be related to each other. The allele frequencies of CYP2D6*1, *2, *10, *19, and *41 in the 130 participants analyzed were 25.0%, 12.7%, 51.5%, 4.2%,and 2.3%, respectively. These findings were similar to the data presented in previous studies (Lee et al., 2009; Yoo et al., 2011). The CYP2D6*3, *7, *9, *11, *14, *20, *44, and *56 alleles were not detected in any of the participants included in the current study. Phenotype extrapolation from *CYP2D6* genotype resulted in 71 (54.6%) and 59 (45.4%) patients classified into the NM and IM groups, respectively. Frequencies of each *CYP2D6* genotype and the corresponding inferred phenotype are presented in **Table 2**.

**Table 2:** Frequencies of *CYP2D6* phenotypes and corresponding *CYP2D6* diplotypes identified in the present study.

*CYP2D6 Phenotype*	*CYP2D6 diplotype*	Activity Score	*N*	*Frequency*
Normal metabolizer(NM)	*1/*1	2	13	10.00%
	*1/*10	1.25	27	20.77%
	*1/*2	2	5	3.85%
	*1/*41	1.5	1	0.77%
	*1/*49	1.5	1	0.77%
	*2/*10	1.25	15	11.54%
	*2/*2	2	5	3.85%
	*2/*41	1.5	3	2.31%
	*43/*43	2	1	0.77%
	All		71	54.62%
Intermediate metabolizer(IM)	*1/*19	1	5	3.85%
	*10/*10	0.5	41	31.54%
	*10/*19	0.25	5	3.85%
	*10/*41	0.75	1	0.77%
	*19/*41	0.5	1	0.77%
	*4/*10	0.5	1	0.77%
	*49/*49	1	1	0.77%
	*6/*10	0.25	3	2.31%
	*6/*17	0.5	1	0.77%
	All		59	45.38%

For each CYP2D6 diplotype, the resulting allelic score was also included.

**Figure 1:**
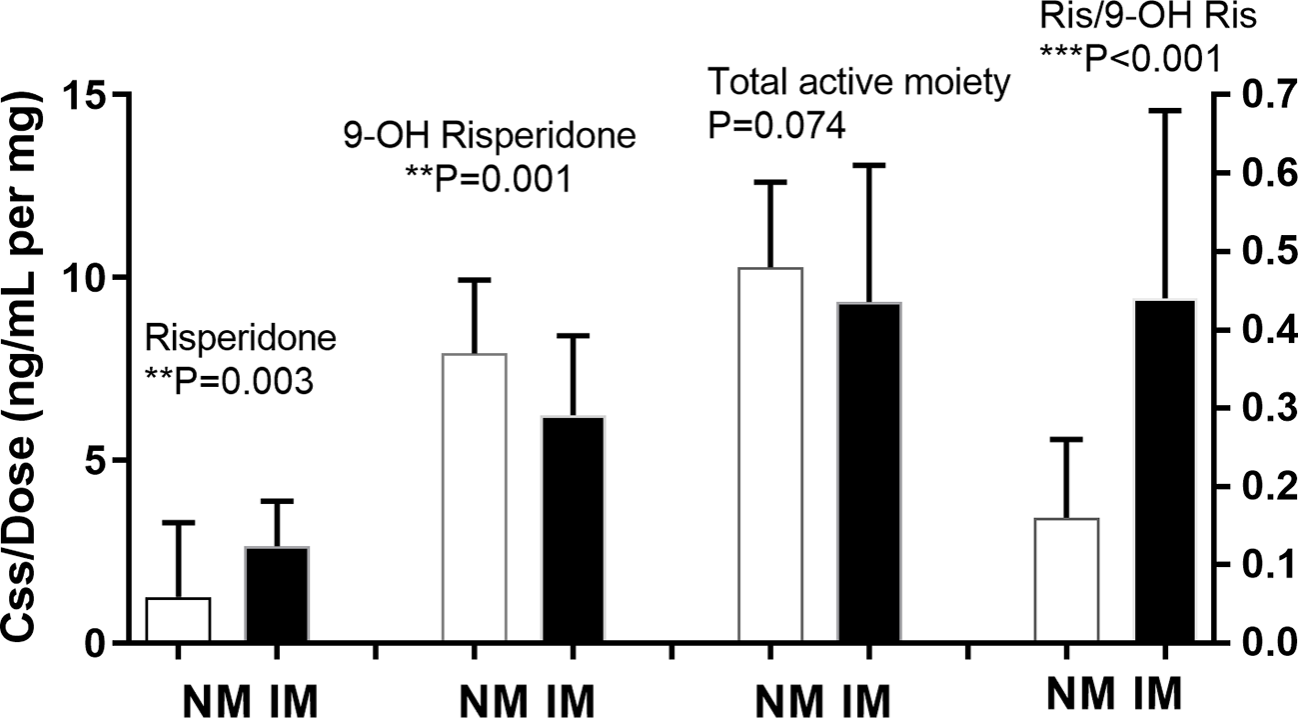
Relationship between *CYP2D6* predicted phenotype and risperidone level, 9-hydroxyrisperidone level, total active moiety level, and risperidone/9-hydroxyrisperidone level. Bars represent the median (interquartile range). The metabolic ratio was plotted on the right Y-axis. NM, normal metabolizer; IM, intermediate metabolizer; RIS, risperidone; 9-OH RIS,9-hydroxyrisperidone; RIS/OHRIS, risperidone/9-hydroxyrisperidone ratio. **means p < 0.01, ***means p < 0.001.

Of the 130 participants, for 38 there were no plasma risperidone levels available. There was no significant difference between the demographic characteristics of these missing participants and the other 92 participants (data not shown). The median daily baseline dosages of risperidone were 6mg and 5mg in the NM and IM group, respectively, as shown in **Table 3**, with no significant between-group difference(p=0.275). The nonparametric test for pairwise comparisons between groups identified significant differences between the NM and IM groups for dose-adjusted risperidone concentration (p=0.003), 9-hydroxyrisperidone concentration (p=0.001), and risperidone/9-hydroxyrisperidone ratio (p<0.001), as shown in **Figure 1** and **Table 3**. As CYP2D6*10 has a high frequency in Asian people and is associated with decreased enzyme activity, an additional head to head comparison was made between *1/*1, *1/*10, and *10/*10 patients for risperidone pharmacokinetic parameters (**Table S1-3** and **Figure S1**).

**Table 3:** The relationship between *CYP2D6* predicted phenotype and risperidone daily dose(mg/d), and dose-adjusted concentrations of risperidone, 9-hydroxyrisperidone, total active moiety, and risperidone/9-hydroxyrisperidone ratio (n =92).

*CYP2D6 predicted phenotype*	*no. n=92(%)*	*CYP2D6 genotype*	*risperidone daily dose (mg/d)*	*risperidone level/Dose* (ng/ml per mg)	*9-hydroxy risperidone/Dose (ng/ml per mg)*	*Total active moiety/Dose* (ng/ml per mg)	*risperidone/9-hydroxy risperidone ratio*
NM	43(46.7)	*1/*1,*1/*10,*1/*2,*1/*41,*1/*49,*2/*10,*2/*2,*2/*41,*43/*43	6(4-7)	1.25(0.62-20.64)	7.93(2.31-25.65)	10.38(5.73-29.01)	0.16(0.07-5.83)
IM	49(53.3)	*1/*19,*10/*10,*10/*19,*10/*41,*19/*41,*4/*10,*49/*49,*6/*10,*6/*17	5(2-6)	2.64(0.67-14.23)	6.23(1.65-17.04)	8.98(2.37-24.6)	0.44(0.08-5.12)
*p* value			0.275	0.003*	0.001*	0.074	<0.001*

*P-value < 0.05. Statistical significance was calculated by Mann-Whitney U test.

NM, normal metabolizer; IM, intermediate metabolizer. Data was represented as the median(range).

Among the 130 patients, 77 patients were prescribed benzhexol to alleviate extrapyramidal-related symptoms, and 73 patients were given benzodiazepines, such as clonazepam or alprazolam, as hypnotics. No other potential *CYP* inhibitors were taken.

### The Relationship Between *CYP2D6* Predicted Phenotype and Plasma Concentrations of Risperidone and Its Metabolites (Pooled and Meta-Analysis)

**Figure 2:**
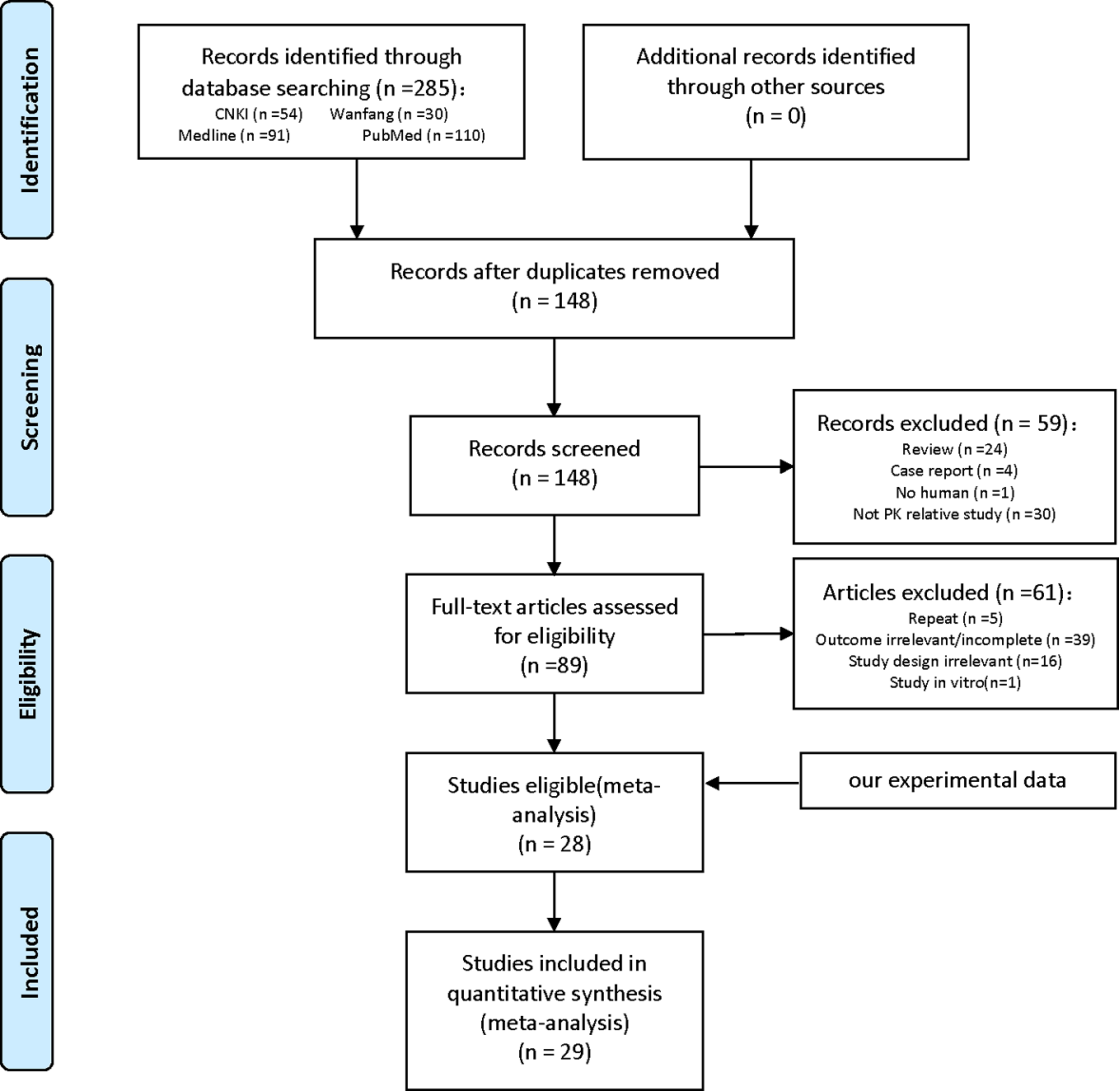
PRISMA flow chart of the study selection.

A systematic review of the literature identified 28 independent studies that met the pre-defined search criteria. Twenty-nine studies were included in our meta-analysis, with a total sample size of 2624 participants (**Figure 2**).

**Figure 3:**
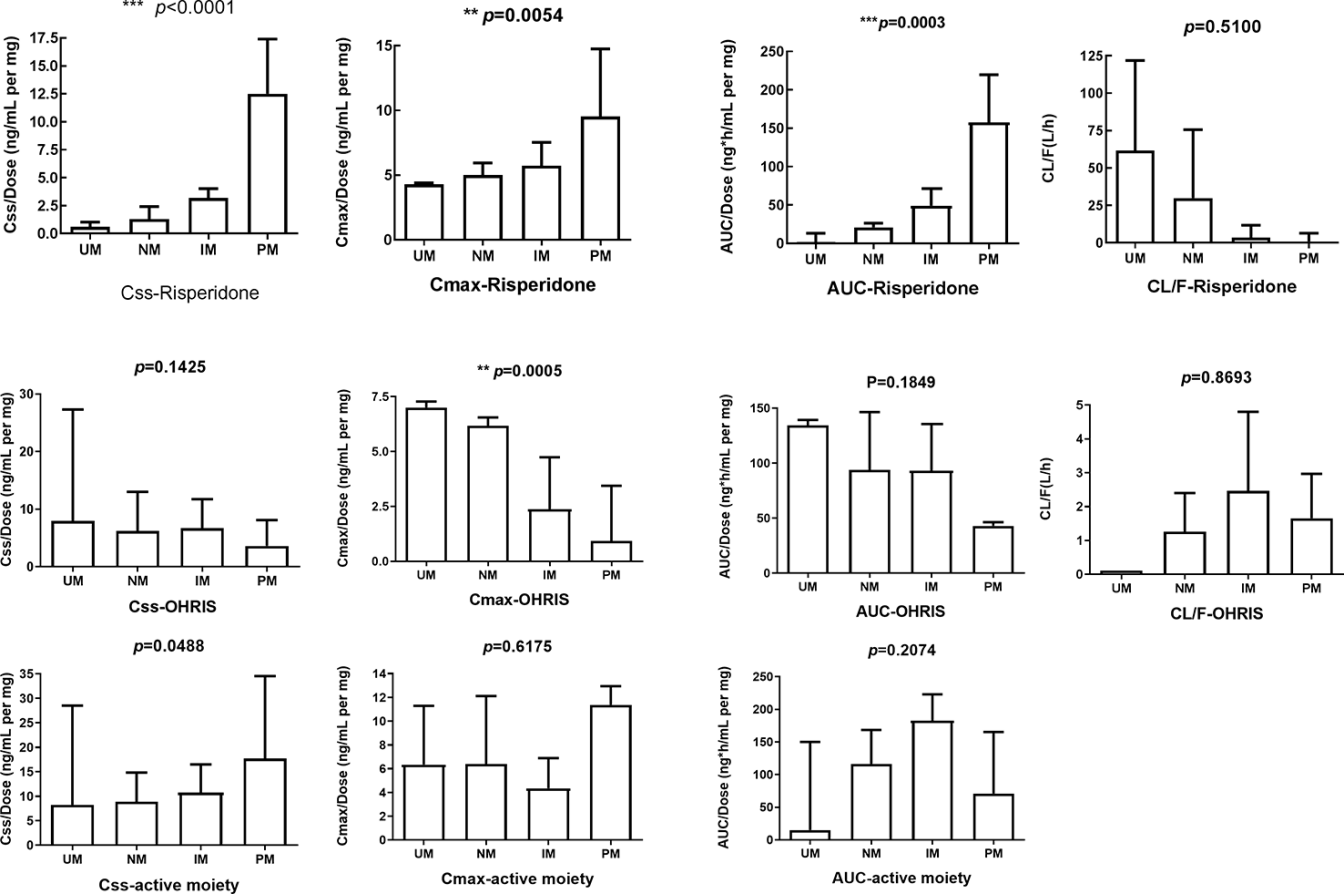
Effects of *CYP2D6* metabolizer phenotype on selected pharmacokinetic parameters. All parameters, except apparent oral clearance, were dose-adjusted. The first row represents pharmacokinetic parameters for risperidone, the second for 9-hydroxyrisperidone, and the third for active moiety of risperidone. There was only one paper reporting the CL/F of the active moiety of risperidone, therefore, this was not pooled. Css, Steady state dose-normalized; Cmax, peak dose-normalized plasma; AUC, dose-normalized area under the plasma concentration; CL/F, apparent oral clearance. Error bars represent standard deviations. **means p < 0.01, ***means p < 0.001.

The pooled pharmacokinetic data are shown in **Table 4** and **Figure 3**. For risperidone, significant differences were observed among the four phenotype groups for the following parameters: the steady state concentration [Css, p<0.0001], peak risperidone concentration [Cmax, p=0.0054], and area under the curve [AUC, p=0.0003], using a Kruskal-Wallis test. No significant between group difference was observed for the oral clearance [CL/F, p=0.5100]. For 9-OH-RIS, a significant difference was found only in the Cmax [p=0.0005]. For the total active moiety, a significant difference was found in the Css[p=0.0488], with no significant difference for the Cmax or AUC (there was only one paper reporting the CL/F of the active moiety of risperidone, therefore it could not be pooled). Subgroup analyses were conducted to examine whether the effects of *CYP2D6* phenotype differed between Asian and white people. However, owing to limited data available for Cmax, AUC, and CL/F, subgroup analysis by ethnicity was conducted only for Css. For pooled analysis of the pharmacokinetics of 9-OH-RIS and the activity moiety stratified by *CYP2D6* phenotype, see **Supplement 2** (**Table S2** and **Figure S2**).

**Table 4:** Pooled analysis for pharmacokinetics of risperidone, 9-hydroxyrisperidone, and the activity moiety stratified by the *CYP2D6* phenotype.

	*Css/dose(ng/mL/mg)*				*Cmax(ng/mL)/Dose*					AUC (ng*h/mL)/Dose					*CL (L/h)/Dose*			
	*N*	*25% percentile*	*Median*	*75% percentile*	*N*	*25% percentile*	*Median*	*75% percentile*		*N*		*25% percentile*	*Median*	*75% percentile*	*N*	*25% percentile*	*Median*	*75% percentile*
**Risperidone**																		
**UM**	35	0.27	0.6	1.03	19	4.28	4.3	4.4		19		10.28	10.7	13.28	12	1.64	61.75	121.9
**NM**	957	0.62	1.29	2.41	141	4.43	5	5.94		161		15.08	20.94	26.37	90	0.65	29.82	75.58
**IM**	487	2.47	3.19	4.02	91	5.31	5.74	7.54		97		46.8	52.58	76.33	60	1.15	8.44	33.77
**PM**	84	6.27	12.5	17.4	21	8.82	9.53	14.75		23		68.63	157.5	219.7	13	0.1	0.9	6.43
***p-value***			<0.0001*				0.0054*						0.0003*				*0.51*	
**9-hydroxyrisperidone**																		
**UM**	30	4.1	7.95	27.3	19	6.97	7	7.28		19		97	134.3	139.3	19	0.11	0.11	0.11
**NM**	890	5.35	6.21	13.01	161	3.76	6.18	6.56		161		35.37	93.84	146.5	161	0.12	1.26	2.4
**IM**	461	6.17	6.71	11.74	97	1.96	2.38	4.74		97		31.31	93.04	135.5	97	0.13	2.47	4.8
**PM**	74	2.32	3.6	8.1	23	0.89	0.93	3.44		23		15.69	42.97	46.32	23	0.34	1.66	2.97
***p-value***			*0.1425*				0.0005***						*0.1849*				*0.2322*	
**Active moiety**																		
**UM**	54	6.753	8.24	28.5	19	1.4	6.35	11.29		19		11.28	15	150				
**NM**	1538	6.66	8.875	14.82	161	1.6	6.39	12.12		161		54.82	116.4	168.5				
**IM**	562	9.315	10.75	16.51	97	1.8	4.35	6.89		97		60.89	182.7	223.1				
**PM**	142	11.31	17.69	34.56	23	9.8	11.37	12.94		23		46.65	71	165.4				
***p-value***			0.0488*				*0.6175*						*0.2074*					

*P-value < 0.05. Statistical significance was calculated by the Kruskal-Wallis test.

A meta-analysis of the data presented in **Table 4** demonstrated a significant effect in all pharmacokinetic (PK) parameters except for clearance. Because the normal metabolizers (NM) represented the majority of the population, NM was set as the reference group. When comparing the total active moiety of risperidone between ultrarapid metabolizers (UM) and NMs, a significant difference was found in Css/dose (mean difference[90% CI]: -4.12[-7.50, -0.75], p=0.02). Moreover, when comparing intermediate metabolizers(IM) and NM, significant differences were found in Css/dose (mean difference[90% CI]:-2.48[-3.70,-1.27], p<0.0001) and AUC/dose (mean difference[90% CI]:-20.68[-23.50,-17.86, p<0.00001). In addition, when comparing poor metabolizers (PM) with NM, the same trend was observed and nominally significant differences were found in the Css/dose (mean difference[90% CI]:- 7.17[-13.48,-0.87], p=0.03) and AUC/dose (mean difference[90% CI]:-19.85[-36.69,-3.01], p=0.02). For Forest plots, see **Figures S3-1–S3-9**).

### Impact of *CYP2D6* Polymorphisms on Dosing of Risperidone

Information on the four *CYP2D6* phenotypic groups for all human studies found for risperidone is shown in **Table S4** (Supplement 4), with the percent dose adjustment calculated based on pharmacokinetic parameters (oral clearance, area under the concentration time curve, and concentration at steady state) observed by phenotype group. For the UM group, extrapolation was performed assuming a linear gene–dose effect according to the calculation methods given above. If possible, data on the active moiety of the drug (sum of parent drug and active metabolite) after multiple dosing were taken, and in cases where more studies existed, mean dose adjustments were calculated. Good concordance of the quantitative effects on pharmacokinetic parameters existed between various studies. If the concentration of the active moiety was known, dose recommendations were based on this. Thus, in summary for Asian people, we suggest the risperidone dose should be reduced by 26% for PM, and increased by 30% for UM, while for white people, for PMs the dose should be reduced by 45% and for UMs increased by 33%.

## Discussion

Risperidone is an active compound that is mainly metabolized in the liver by cytochrome P450 enzymes such as *CYP2D6*. An active metabolite in the main hydroxylation pathway is 9-hydroxyrisperidone. Another minor metabolic pathway involves *N*-dealkylation (Fang et al., 1999). In a previous *in vitro* study, three CYP enzymes were identified, *CYP2D6*, CYP3A4, and CYP3A5, which showed risperidone to 9-hydroxyrisperidone metabolizing activities, at 7.5, 0.4, and 0.2 pmol pmol^–1^ CYP min^–1^, respectively (Fang et al., 1999). In *in vivo* studies, Yasui- Furukori et al. (Yasui-Furukori et al., 2001) demonstrated that *CYP2D6* played a predominant role in the formation of (+)-9-hydroxyrisperidone, while CYP3A4 appeared to be primarily involved in the formation of (-)-9-hydroxyrisperidone. These findings were confirmed by using inhibitors of CYP2D6 (quinidine) and CYP3A4 (ketoconazole) to inhibit the formation of 9-hydroxyrisperidone (Fang et al., 1999). Thus, 9-hydroxyrisperidone is the major active metabolite of risperidone, and CYP2D6 is the major enzyme involved in its formation, although CYP3A4 also seems to contribute.

In this study, we first investigated the influence of *CYP2D6* polymorphism on the pharmacokinetics of risperidone in 130 Chinese outpatients. According to results presented previously (Johansson et al., 1994; Yoo et al., 2011), Asian populations show a high frequency (about 50%) for the CYP2D6*10 allele and a low frequency of nonfunctional *CYP2D6* alleles. In our study, the allele frequency of CYP2D6*10 was 51.5%, which is similar to that presented previously. As shown in a meta-analysis (Gaedigk et al., 2017), over 99% of Asians are normal metabolizers (66.5%) and intermediate metabolizers (32.5%). In our study, 71 out of 130 (54.62%) patients were NM and 59 out of 130 (45.38%) were IM, with no participants in the PM or UM groups. However, it should be noted that we did not use a method of directly identifying Ums but rather inferred these from array data, and therefore it is possible that there were patients who were UMs or with other structural variants of *CYP2D6* that were not identified in our sample.

For risperidone pharmacokinetics in the Chinese sample, significant differences were observed between the NM and IM groups for the dose-adjusted concentrations of risperidone (p=0.003), 9-hydroxyrisperidone (p=0.001), and the risperidone/9-hydroxyrisperidone ratio (p<0.001). Our meta-analysis results were consistent with this. In risperidone-treated patients, the RIS/9-OH-RIS concentration ratio is an index of *CYP2D6* activity and the concentration-to-dose (C/D) ratio, in which C includes RIS+9-OH-RIS, is an index of the active moiety and its total clearance from the body (de Leon et al., 2010). Our results indicated that the NM and IM groups had different *CYP2D6* activity but did not show significant differences in total clearance from the body. The pooled analysis and meta-analysis provide more robust data regarding the effects of different *CYP2D6* metabolizer phenotypes on the pharmacokinetics of risperidone in white and Asian people.

The US Food and Drug Administration (FDA) (https://www.accessdata.fda.gov/scripts/cder/daf/index.cfm?event=overview.process&varApplNo=210655) appears to have considered that, as the active moiety was the sum of risperidone and 9-hydroxyrisperidone, and CYP2D6 metabolizer status would be predicted to merely change the ratio between these two but not the total of the two, there was no need for a recommendation for patients to undergo cytochrome P450 (CYP) PGx testing when initiating risperidone. Similarly, the Royal Dutch Pharmacists Association - Pharmacogenetics Working Group (DPWG) 2018 update did not recommend that any action was required for this gene-drug interaction (https://www.pharmgkb.org/chemical/PA451257/guidelineAnnotation/PA166104943) Although Swen et al. (2011) concluded that there were “insufficient data to allow calculation of dose adjustment,” they also suggested selection of an alternative drug, vigilance for adverse drug events, and/or adjustment of doses according to clinical response for patients who were *CYP2D6* poor metabolizers, intermediate metabolizers, or ultrarapid metabolizers (Swen et al., 2011). Subsequent works (Stingl and Viviani, 2015; Jukic et al., 2019) showed that patients in the PM or UM groups had a significantly higher rate of switching from risperidone to another medication. Jukic et al. (Jukic et al., 2019) analyzed data from 1288 patients treated with risperidone and found that CYP2D6 IMs and PMs had 1.4- and 1.6-fold higher exposure, respectively, to the active moiety for risperidone (i.e., risperidone plus 9-hydroxyrisperidone). The rate of switching to another medication was also higher in UMs and PMs than in NMs. Roos van Westrhenen et al. (2020) recommended reduction of risperidone dose by 33% in PMs and IMs on the basis of the results of Jukic et al. (2019). Our meta-analytic data support dose adjustments for risperidone based on CYP2D6 genotype for white people, and also suggest a greater reduction (45%) for PMs. This data also extends to Asian populations. We have computed dose adjustments as follows. In Asian populations, for PMs reduce the recommended dose by 26% and for UMs increase the dose by 30%. In white people, for PMs reduce the recommended dose by 45% and for UMs increase it by 33%.

### Limitations and Future Directions

As mentioned above, a limitation of our study was the method of derivation of CYP2D6 copy number. How UMs and those with structural variants are assessed is likely to be a limitation common to previous studies in this field. Therefore, the dose recommendation for Asian people who are UMs should be considered provisional, pending replication.

In this study, we also measured the clinical response for the 130 patients by Positive and Negative Syndrome Scale (PANSS) at the baseline and the end of two weeks, four weeks, and six weeks after taking the assigned antipsychotic medication. We did not observe any association between *CYP2D6* polymorphism and the PANSS percent change in our sample of 130 Asian patients with schizophrenia (t=0.561, p=0.576 at 2 weeks; t=0.196, p=0.845 at 4 weeks; t=-0.129, p=0.898 at six weeks).

In this study, dose adjustments were computed assuming a linear relationship between relative dose adjustments and relative changes of pharmacokinetic parameters observed in the phenotype groups. While this may not be valid (Jukic et al., 2019), as we did not observe any UMs, the assumption of linearity could not be tested.

In our pooled analysis, we incorporated pharmacokinetic parameters, whether from a single-dose study (SD) data or from multiple dosing (MD), as well as from healthy volunteers or patients. Given that saturable pharmacokinetics, irreversible enzyme blockade, and enzyme up- or downregulation might change the outcome under multiple dosing or under different physical conditions, it is inappropriate for data from single-dose experiments to be extrapolated to long-term drug therapy, or, likewise, to extrapolate data from healthy individuals to patients (Kirchheiner et al., 2004). Several factors affect risperidone dosage, including patient-related factors (i.e. gender, age, ethnicity, and body mass index (BMI)), factors related to psychiatric illness (i.e. acute vs. chronic phase, illness severity) (Gardner et al., 2010) and concomitant administration of medication (i.e. *CYP2D6* inhibitors, such as fluoxetine or paroxetine, and *CYP3A4* inducers such as carbamazepine or phenytoin) (Spina and de Leon, 2015). Moreover, multigenic interactions and gene-environment interactions will have to be considered. Therefore, in the future, haplotypic analyses and multigenetic analyses with large sample sizes, and high-quality phenotypic data should be included. Furthermore, the raw data in our meta-analysis, presented as the median (range), were used to infer the mean (SD), and in some cases the units had to be converted for data harmonization. We acknowledge that this conversion might result in some measurement bias. For CYP2D6 phenotypic grouping, in a previous study, African‐American subjects with CYP2D6*1/*2 and *2/*2 genotypes had significantly lower activity compared with white people. It is also possible that a subgroup of CYP2D6*2 alleles are associated with reduced enzyme activity (Wang et al., 2014; Wang et al., 2015). A potential difference in activity of the *2 allele between Asian and white people might contribute to some of the differences among the enzyme activity of these ethnic groups. This might also result in a degree of dose adjustment being required in CYP2D6 phenotypes derived from CYP2D6*2.

Further research aiming to address the limitations that we have outlined above, particularly in regard to the UM group, is indicated. In order to implement routine pre-treatment genetic testing, additional parameters would need to be considered, including the effects of concomitant *CYP2D6* inhibiting medications and CYP3A inducers, clinical utility, and cost-benefit analyses. In regard to clinical utility, we did not observe any association between *CYP2D6* polymorphism and clinical response in our sample of 130 Asian patients with schizophrenia (as measured by the PANSS).

## Data Availability Statement

The original contributions presented in the study are publicly available. This data can be found here: https://doi.org/10.18170/DVN/MQB2Y3.

## Ethics Statement

This study was carried out in accordance with the recommendations of the institutional ethics review boards, Peking University Sixth Hospital with written informed consent from all subjects. All subjects gave written informed consent in accordance with the Declaration of Helsinki. The protocol was approved by the institutional ethics review boards, Peking University Sixth Hospital.

## Author Contributions

YC and WY presented the ideas. YC and YS developed the theory and performed the computations. HY, LW, and TL collected the samples and carried out the experiment. DZ and WY supervised the project.

## Funding

The study was funded by National Key R&D Program of China (2016YFC1307000, 2017YFC1311100); National Natural Science Foundation of China (81825009, 81901358, 81221002); Peking University Clinical Scientist Program supported by “the Fundamental Research Funds for the Central Universities”(BMU2019LCKXJ012); Academy of Medical Sciences Research Unit (2019-I2M-5-006); PKUHSC-KCL Joint Medical Research (BMU2020KCL001); Beijing Science and Technology Commission (D171100007017002).

## Conflict of Interest

The authors declare that the research was conducted in the absence of any commercial or financial relationships that could be construed as a potential conflict of interest.
