# Supplementary material for: CYP2D6 Genotype-Based Dose Recommendations for Risperidone in Asian People
Source: Front Pharmacol. 2020 Aug 4;11:936. doi: 10.3389/fphar.2020.00936 (PMC7417932; doi:10.3389/fphar.2020.00936)
Supplement: Supplementary file 1 [file DataSheet_1.docx]

**Supplement 1**

| **Table S1-1.** Allele frequencies of the 17 single nucleotide polymorphisms genotyped in the present study. | | | | |  |
| --- | --- | --- | --- | --- | --- |
| **SNP** | ***CYP2D6* Allele** | **Variants** | **MAF 1000 Genomes** | **MAF Present Study** | **Call Rate per marker** |
| rs5030655 | **6* | 1708delT | 0 | 0.032 | 100 |
| rs16947 | **2,*11,*17,*19,*41* | 886C>T | 0.165 | 0.196 | 100 |
| rs28371706 | **17* | 1022C>T | 0 | 0.013 | 88.46 |
| rs3892097 | **4* | 1847G>A | 0.0049 | 0.004 | 100 |
| rs72549353  (rs758320086 ) | **19* | 2540delAACT | ? | 0.262 | 93.85 |
| rs1065852 | **4,*10,*36, *37, *47,*49,*51,*52,*54,*57,*64,*65,*69,*72,*87,*94,*95,*99,*100,*101,*114,*117,*132* | 100C>T | 0.6019 | 0.538 | 100 |
| rs1135822 | **49* | 1612T>A | ? | 0.011 | 100 |
| rs28371725 | **41* | 2989G>A | 0.034 | 0.031 | 100 |
| rs28371696 | **43* | 77G>A | 0 | 0.011 | 100 |
| rs147960066  (rs72549347) | **56* | 3202C>T | 0 | 0 | 100 |
| rs201377835 | **11* | 882G>C | 0 | 0 | 100 |
| rs72549349 | **44* | 2951G>C | ? | 0 | 100 |
| rs72549354 | **20* | 1977_1978insG | ? | 0 | 100 |
| rs35742686 | **3* | 2550delA | 0 | 0 | 100 |
| rs5030865 | **14* | 1759G>A | 0.0049 | 0 | 100 |
| rs5030656 | **9* | 2616delAAG | 0 | 0 | 100 |
| rs5030867 | **7* | 2936A>C | 0 | 0 | 100 |
| ? There is no frequency data. | | |  |  |  |
| Abbreviations: SNP, single nucleotide polymorphism; MAF, minimum allele frequency. | | | | |  |

| **Table S1-2.** Frequencies of the CYP2D6 alleles identified in the present study. The variants used to define each CYP2D6 allele, its activity and its activity score were included. | | | | |
| --- | --- | --- | --- | --- |
| ***CYP2D6* Allele** | ***CYP2D6* Variants** | **Enzyme Activity** | **Activity Score** | **Allele Frequency** |
| *CYP2D6*1* |  | Normal | 1 | 0.250 |
| *CYP2D6*2* | rs16947_T | Normal | 1 | 0.127 |
| *CYP2D6*4* | rs3892097_A | Null | 0 | 0.004 |
| *CYP2D6*6* | rs5030655_delT | Null | 0 | 0.015 |
| *CYP2D6*10* | rs1065852_T | Diminished | 0.25 | 0.515 |
| *CYP2D6*17* | rs28371706_T; rs16947_T | Diminished | 0.5 | 0.004 |
| *CYP2D6*19* | rs72549353_delAACT; rs16947_T | Null | 0 | 0.042 |
| *CYP2D6*41* | rs28371725_A; rs16947_T | Diminished | 0.5 | 0.023 |
| *CYP2D6*43* | rs28371696_A | Normal | 1 | 0.008 |
| *CYP2D6*49* | rs1135822_A; rs1065852_T | Diminished | 0.5 | 0.012 |

**Table S1-3.** The relationship between CYP2D6 *1/*1, *1/*10, *10/*10 phenotypes and risperidone daily dose(mg/d), risperidone, 9-hydroxyrisperidone, total active moiety and risperidone/9-hydroxyrisperidone ratio.

| CYP2D6 predicted phenotype | N | 9-OH risperidone/Dose (ng/ml per mg) | | | risperidone level/Dose (ng/ml per mg) | | | Total active moiety/Dose (ng/ml per mg) | | | risperidone/9-OH risperidone ratio | | |
| --- | --- | --- | --- | --- | --- | --- | --- | --- | --- | --- | --- | --- | --- |
|  |  | 25% | median | 75% | 25% | median | 75% | 25% | median | 75% | 25% | median | 75% |
| *1/*1 | 13 | 5.33 | 6.64 | 7.26 | 0.85 | 2.16 | 3.67 | 7.61 | 10.50 | 13.54 | 0.13 | 0.24 | 0.37 |
| *1/*10 | 27 | 5.10 | 7.10 | 9.44 | 0.78 | 0.89 | 1.20 | 7.92 | 9.89 | 11.17 | 0.09 | 0.11 | 0.17 |
| *10/*10 | 41 | 4.00 | 5.49 | 7.66 | 1.92 | 2.74 | 3.85 | 6.18 | 8.61 | 10.26 | 0.33 | 0.44 | 0.65 |
| *p* value |  | *p=0.080* | | | *p=0.001^#^* | | | *p=0.314* | | | *p<0.001*^#^ | | |

*P-value < 0.05. Statistical significance was calculated by Kruskal-Wallis test. #Pairwise comparisons indicated that there was significant difference between *1/10 and *10/*10 (p<0.001,after Bonferroni adjustment).

**Figure S1.** Relationship between CYP2D6 *1/*1, *1/*10, and *10/*10 patients and risperidone level, 9-hydroxyrisperidone level, total active moiety level and risperidone/9-hydroxyrisperidone level. Bars represent median (interquartile range). The metabolic ratio was plotted on the right Y-axis. Ris, risperidone; 9-OH Ris,9-hydroxyrisperidone; Total active moiety= risperidone + 9-hydroxyrisperidone; Ris/9-OH Ris, risperidone/9-hydroxyrisperidone ratio.

**Supplement 2:**

Subgroup analyses were conducted only for Css in Asians or Europeans separately.

**Table S2.** Pooled analysis of steady state concentration of risperidone stratified by CYP2D6 phenotype in Asians and Europeans separately.

|  | **Css/dose(ng/mL/mg) in Asians** | | | | | | | | | | | |
| --- | --- | --- | --- | --- | --- | --- | --- | --- | --- | --- | --- | --- |
|  | **Risperidone** | | | | **9-OH risperidone** | | | | **Active moiety** | | | |
|  | **N** | **25% percentile** | **Median** | **75% percentile** | **N** | **25% percentile** | **Median** | **75% percentile** | **N** | **25% percentile** | **Median** | **75% percentile** |
| **UM** | 4 | 0.00 | 0.00 | 0.00 | 4 | 45.58 | 45.58 | 45.58 | 4 | 52.48 | 52.48 | 52.48 |
| **NM** | 470 | 0.42 | 1.48 | 2.21 | 474 | 4.96 | 5.58 | 7.56 | 474 | 6.42 | 7.01 | 11.15 |
| **IM** | 179 | 1.99 | 3.26 | 4.02 | 173 | 6.16 | 6.58 | 7.84 | 183 | 7.94 | 10.05 | 14.50 |
| **PM** | 8 | 5.56 | 5.92 | 6.27 | 8 | 0.21 | 1.39 | 2.56 | 8 | 5.79 | 7.32 | 8.84 |
| **p-value** | 0.0048** | | | | <0.0001**** | | | | <0.0001**** | | | |

|  | **Css/dose(ng/mL/mg) in Europeans** | | | | | | | | | | | |
| --- | --- | --- | --- | --- | --- | --- | --- | --- | --- | --- | --- | --- |
|  | **Risperidone** | | | | **9-OH risperidone** | | | | **Active moiety** | | | |
|  | **N** | **25% percentile** | **Median** | **75% percentile** | **N** | **25% percentile** | **Median** | **75% percentile** | **N** | **25% percentile** | **Median** | **75% percentile** |
| **UM** | 31 | 0.35 | 0.60 | 1.09 | 26 | 3.95 | 7.16 | 15.53 | 50 | 5.918 | 7.83 | 11.26 |
| **NM** | 487 | 0.78 | 1.10 | 2.61 | 416 | 6.15 | 8.32 | 23.00 | 1064 | 7.215 | 9.23 | 18 |
| **IM** | 308 | 2.48 | 3.19 | 6.60 | 288 | 6.10 | 12.16 | 21.75 | 379 | 9.222 | 11.93 | 16.37 |
| **PM** | 76 | 9.79 | 13.85 | 17.70 | 66 | 2.42 | 3.85 | 8.30 | 134 | 12.81 | 17.69 | 26.86 |
| **p-value** | 0.0004*** | | | | 0.2234 | | | | 0.0144* | | | |

*P-value < 0.05. Statistical significance was calculated by Kruskal-Wallis test.


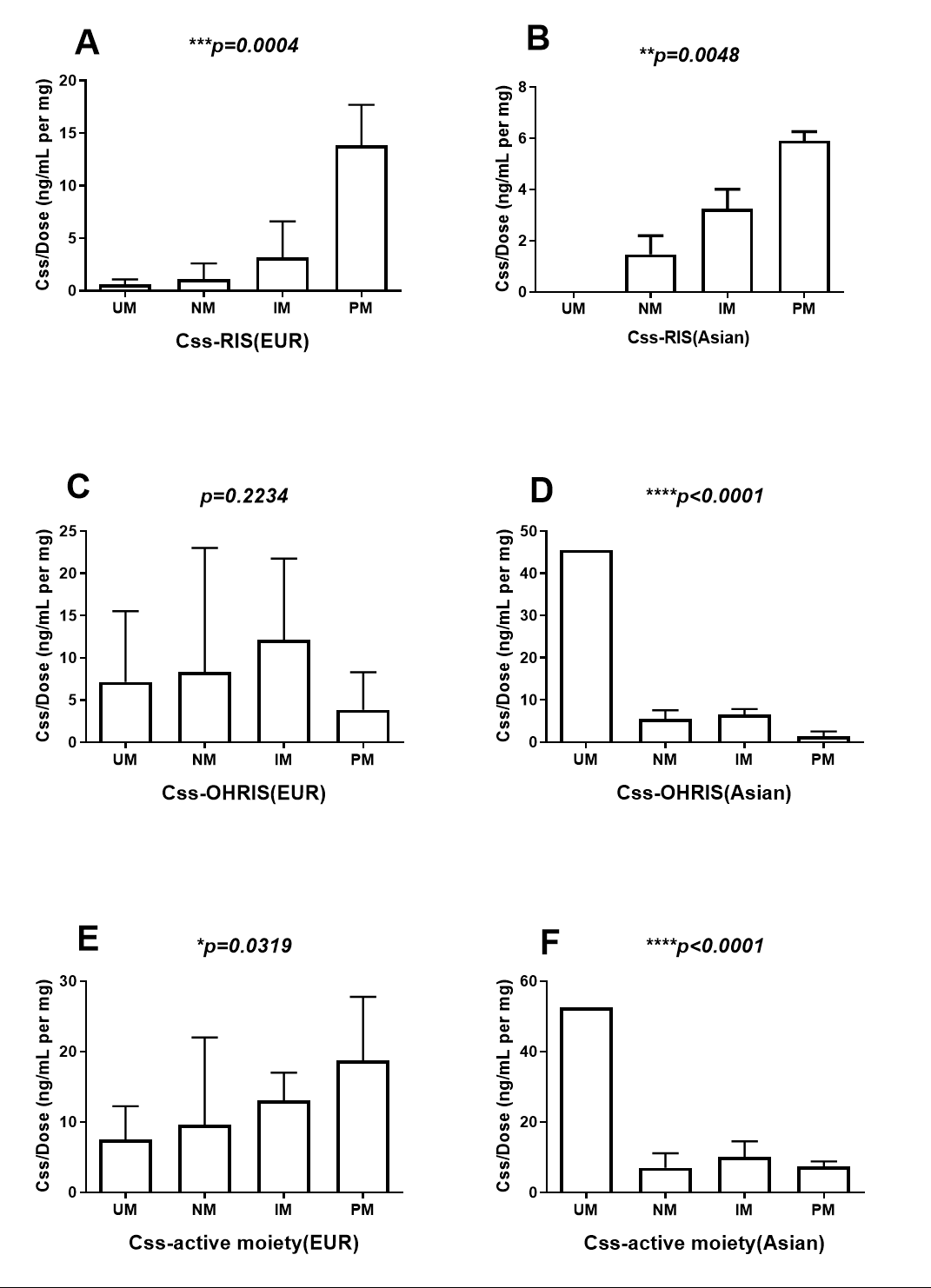


**Figure S2.** A-F. Effects of CYP2D6 metabolizer phenotype on Steady state dose-normalized concentration for risperidone, 9-OH risperidone and the total active moiety in Asians and Europeans separately.

**A** Steady state dose-normalized plasma risperidone concentration (Css/dose) in Europeans; **B** Steady state dose-normalized plasma 9-OH risperidone concentration (Css/dose) in Asians; **C** Steady state dose-normalized plasma 9-OH risperidone concentration (Css/dose) in Europeans; **D** Steady state dose-normalized plasma 9-OH risperidone concentration (Css/dose) in Asians; **E** Steady state dose-normalized plasma active moiety(risperidone+9-OH risperidone) concentration (Css/dose) in Europeans; **F** Steady state dose-normalized plasma active moiety(risperidone+9-OH risperidone) concentration (Css/dose) in Asians.

**Supplement 3**


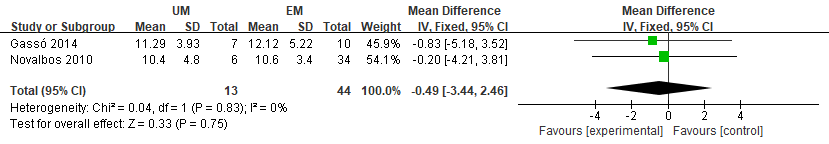


**Figure S3-1.** Forest plot of comparison of risperidone and 9-OH risperidone (Cmax/Dose) between UM and EM group.


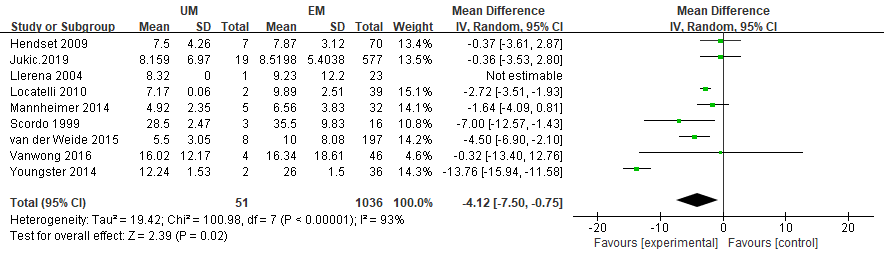


**Figure S3-2.** Forest plot of comparison of risperidone and 9-OH risperidone (Css/Dose) between UM and NM/EM group.


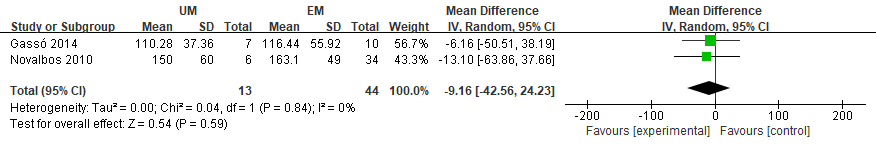


**Figure S3-3.** Forest plot of comparison of risperidone and 9-OH risperidone (AUC/Dose) between UM and NM/EM group.


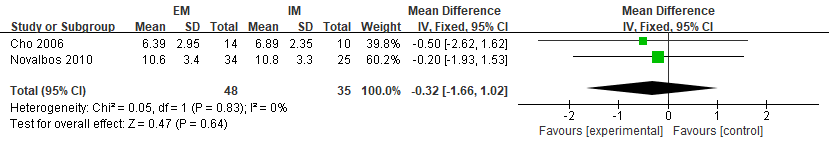


**Figure S3-4.** Forest plot of comparison of risperidone and 9-OH risperidone (Cmax/Dose) between NM/EM and IM group.


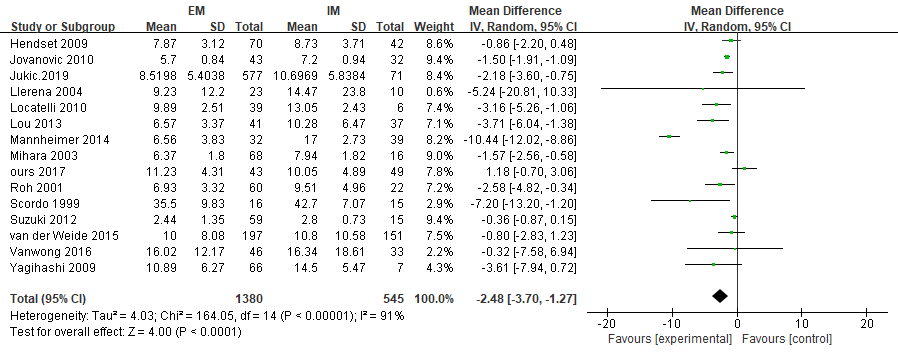


**Figure S3-5.** Forest plot of comparison of risperidone and 9-OH risperidone (Css/Dose) between NM/EM and IM group.


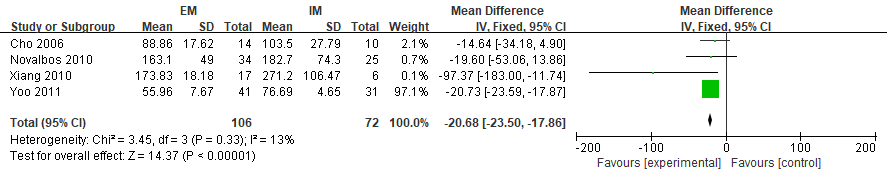


**Figure S3-6.** Forest plot of comparison of risperidone and 9-OH risperidone (AUC/Dose) between NM/EM and IM group.


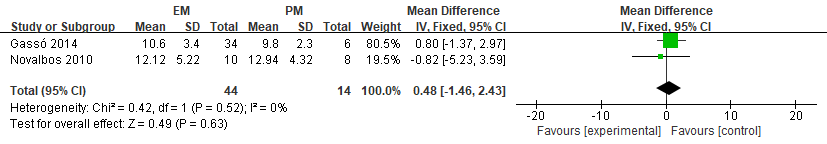


**Figure S3-7.** Forest plot of comparison of risperidone and 9-OH risperidone (Cmax/Dose) between NM/EM and PM group.


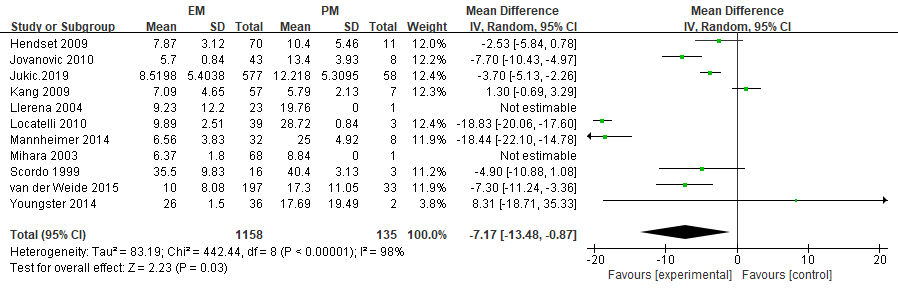


**Figure S3-8.** Forest plot of comparison of risperidone and 9-OH risperidone (Css/Dose) between NM/EM and PM group.


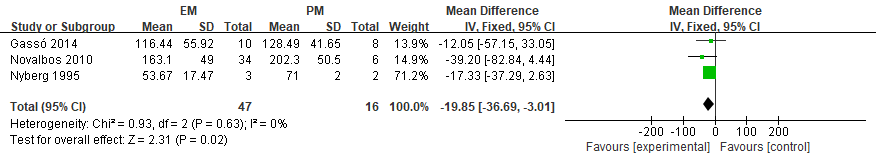


**Figure S3-9.** Forest plot of comparison of risperidone and 9-OH risperidone (AUC/Dose) between NM/EM and PM group.

**Supplement 4**

**Table S4.** Dose adjustments of risperidone based on empirical pharmacokinetic parameters measured in CYP2D6 phenotypes.

| ***Measured*** | ***Ethnic*** | ***Parameter*** | ***N*** | ***UM*** | ***NM*** | ***IM*** | ***PM*** | **References** | | | |
| --- | --- | --- | --- | --- | --- | --- | --- | --- | --- | --- | --- |
|  |  |  | ***UM/NM/IM/PM*** | ***(%)*** | ***(%)*** | ***(%)*** | ***(%)*** |  |  |  |  |
| P/Asian | Korean | Css | 0/60/22/0 | 172.43# | 118.25 | 64.07 | 43.94# | Roh et al., 2001 | | | |
|  | Japan | Css | 0/68/16/1 | 255.67# | 139.13 | 22.58 | 5.96 | Mihara et al., 2003 | | | |
|  | Japan | Css | 0/34/4/0 | 255.66# | 139.10 | 22.54 | 12.26# | Yasui-Furukori et al., 2003 | | | |
|  | Korean | AUC | 0/14/10/0 | 209.74# | 127.61 | 45.47 | 27.66# | Cho and Lee, 2006 | | | |
|  | Japan | Css | 0/66/7/0 | 192.97# | 123.40 | 53.83 | 34.43# | Yagihashi et al., 2009 | | | |
|  | Korean | Css | 0/57//7 | 183.67# | 121.07 | 58.47 | 38.54 | Kang et al., 2009 | | | |
|  | China | AUC | 0/17/6/0 | 248.00# | 137.18 | 26.36 | 14.58# | Xiang et al., 2010 | | | |
|  | Korean | AUC | 0/41/31/0 | 218.15# | 129.71 | 41.27 | 24.54# | Yoo et al., 2011 | | | |
|  | Japan | Css | 0/59/15/0 | 183.81# | 121.11 | 58.40 | 38.48# | Suzuki et al., 2012 | | | |
|  | China | Css | 0/41/37/0 | 209.11# | 127.45 | 45.78 | 27.90# | Lou, 2013 | | | |
|  | Thailand | Css | 4/46/33/0 | 243.62 | 136.09 | 28.55 | 15.95# | Vanwong et al., 2016 | | | |
|  | China | CSS | 0/43/49/0 | 185.53# | 121.54 | 57.55 | 37.70# |  | | | |
| P/Asian |  |  |  | **213.20** | **128.47** | **43.74** | **26.83** |  | | | |
| P/White | Italian | Css | 3/16/15/3 | 210.41 | 114.77 | 13.02 | 7.26 | Scordo et al., 1999 | | | |
|  | Caucasian | Css | 1/23/10/1 | (304.08 | 110.58 | 31.59 | 5.75) | Llerena et al., 2016 | | | |
|  | Norway | Css | 7/70/42/11 | 143.91 | 111.07 | 51.40 | 20.23 | Hendset et al., 2009 | | | |
|  | Spain | CL | 6/34/25/6 | 84.21 | 111.38 | 56.59 | 8.98 | Novalbos et al., 2010 | | | |
|  | Sweden | Css | 5/32/39/8 | 344.00 | 107.50 | 47.78 | 9.56 | Mannheimer et al., 2014 | | | |
|  | Caucasian | CL | 6/33/24/5 | 166.49 | 111.10 | 55.06 | 8.79 | Cabaleiro et al., 2014 | | | |
|  | Caucasian | AUC | 7/10/0/8 | 190.10 | 108.06 | 55.40# | 37.25 | Gassó et al., 2014 | | | |
|  | Israel | Css | 2/29/7/2 | (141.18 | 105.88 | 105.88 | 11.76) | Youngster et al., 2014 | | | |
|  | Netherland | Css | 8/197/151/33 | 176.87 | 106.12 | 88.43 | 21.22 | van der Weide and van der Weide, 2015 | | | |
|  | Belgian | Css | 1/12/1/3 | 242.62 | 103.98 | 94.94 | 19.15 | Lisbeth et al., 2016 | | | |
| P/White |  |  |  | **194.83** | **109.25** | **57.83** | **16.55** |  |  |  | |
| p(Asian+White) |  |  |  | **205.85** | **120.78** | **49.37** | **22.72** |  | | | |
| P+MT/Asian | Korean | Css | 0/60/22/0 | 139.96# | 110.09 | 80.22 | 63.10# | Roh et al., 2001 | | | |
|  | Japan | Css | 0/68/16/1 | 128.37# | 107.15 | 85.93 | 77.24 | Mihara et al., 2003 | | | |
|  | Korean | AUC | 0/14/10/0 | (119.88# | 105.03 | 90.18 | 79.01#) | Cho and Lee, 2006 | | | |
|  | Japan | Css | 0/66/7/0 | 136.37# | 109.19 | 82.00 | 65.66# | Yagihashi et al., 2009 | | | |
|  | Korean | Css | 0/57//7 | 86.91# | 96.67 | 106.43 | 118.38 | Kang et al., 2009 | | | |
|  | China | AUC | 0/17/6/0 | (157.47# | 114.50 | 71.52 | 52.00#) | Xiang et al., 2010 | | | |
|  | Korean | AUC | 0/41/31/0 | 137.67# | 109.51 | 81.36 | 64.72# | Yoo et al., 2011 | | | |
|  | Japan | Css | 0/59/15/0 | (118.39# | 104.65 | 90.92 | 80.37#) | Suzuki et al., 2012 | | | |
|  | China | Css | 0/41/37/0 | 154.95# | 113.86 | 72.77 | 53.47# | Lou, 2013 | | | |
|  | Thailand | Css | 4/46/33/0 | (59.25 | 106.73 | 86.84 | 73.20#) | Vanwong et al., 2016 | | | |
|  | China | CSS | 0/43/49/0 | (80.14# | 94.94 | 109.74 | 130.01#) |  | | | |
| P+MT/Asian |  |  |  | **130.71** | **107.75** | **84.79** | **73.76** |  | | |  |
| P+MT/White | Italian | Css | 3/16/15/3 | (126.94 | 101.91 | 84.73 | 89.55) | Scordo et al., 1999 | | | |
|  | Caucasian | Css | 1/23/10/1 | (118.90 | 107.27 | 68.37 | 50.08) | Llerena et al., 2016 | | | |
|  | Norway | Css | 7/70/42/11 | (107.61 | 102.55 | 92.45 | 77.61) | Hendset et al., 2009 | | | |
|  | Maribor | CL | 2/39/6/3 | 102.59 | 109.11 | 74.31 | 25.62 | Locatelli et al., 2010 | | | |
|  | Spain | CL | 6/34/25/6 | (98.28 | 98.28 | 98.28 | 78.62) | Novalbos et al., 2010 | | | |
|  | Sweden | Css | 5/31/38/8 | 136.59 | 102.44 | 96.41 | 65.56 | Mannheimer et al., 2014 | | | |
|  | Caucasian | AUC | 7/10/0/8 | (106.65 | 101.00 | 96.03# | 91.53) | Gassó et al., 2014 | | | |
|  | Israel | Css | 2/29/7/2 | (154.17 | 90.72 | 90.72 | 40.13) | Youngster et al., 2014 | | | |
|  | Netherland | Css | 8/197/151/33 | 185.50 | 102.02 | 94.47 | 58.97 | van der Weide and van der Weide, 2015 | | | |
|  | Spain | Css | 2/18/11/3 | (58.48 | 106.32 | 97.46 | 41.04) | Mas et al., 2017 | | | |
|  | Norway | Css | 19/577/71/58 | 108.62 | 104.02 | 82.85 | 72.53 | Jukic et al., 2019 | | | |
| P+MT/White |  |  |  | **133.32** | **104.40** | **87.01** | **55.67** |  | | | |
| P+MT(Asian+White) |  |  |  | **119.78** | **96.73** | **77.89** | **60.48** |  | | | |
| *Extrapolated dose recommendations from studies where differences were statistically not significant are printed in parenthesis. No dose adjustments based on CYP2D6 genotype are recommended based on these studies; the percentages are solely given for completeness of this quantitative meta-analysis. P: Parent drug; MT: metabolite.* | | | | | | | | | | | |
